# A Handy Liquid Metal Based Non-Invasive Electrophoretic Particle Microtrap

**DOI:** 10.3390/mi9050221

**Published:** 2018-05-07

**Authors:** Lu Tian, Lunjia Zhang, Meng Gao, Zhongshan Deng, Lin Gui

**Affiliations:** 1 Key Laboratory of Cryogenics, Technical Institute of Physics and Chemistry, Chinese Academy of Sciences, 29 Zhongguancun East Road, Haidian District, Beijing 100190, China; lutian@mail.ipc.ac.cn (L.T.); zhanglunjia14@mails.ucas.ac.cn (L.Z.); zsdeng@mail.ipc.ac.cn (Z.D.); 2 University of Chinese Academy of Sciences, 19 Yuquan Road, Shijingshan District, Beijing 100039, China; mgao@mail.ipc.ac.cn

**Keywords:** microfluidics, single particle control, microtrap, liquid metal

## Abstract

A handy liquid metal based non-invasive particle microtrap was proposed and demonstrated in this work. This kind of microtrap can be easily designed and fabricated at any location of a microfluidic chip to perform precise particle trapping and releasing without disturbing the microchannel itself. The microsystem demonstrated in this work utilized silicon oil as the continuous phase and fluorescent particles (PE-Cy5, SPHERO^TM^ Fluorescent Particles, BioLegend, San Diego, CA, USA, 10.5 μm) as the target particles. To perform the particle trapping, the micro system utilized liquid-metal-filled microchannels as noncontact electrodes to generate different patterns of electric field inside the fluid channel. According to the experimental results, the target particle can be selectively trapped and released by switching the electric field patterns. For a better understanding the control mechanism, a numerical simulation of the electric field was performed to explain the trapping mechanism. In order to verify the model, additional experiments were performed and are discussed.

## 1. Introduction

Manipulation of a single particle, droplet, or cell is a critical tool for microfluidic analysis in many applications including flow cytometer detection, biochemical analysis, and so on [1,2,3]. Traditionally, particles, droplets, and cells are usually manipulated in groups [4]. However, single particles, droplets, or cells can provide more distinctive information for microfluidic analysis [5,6]. As the methods of manipulating particles, droplets, and cells are sometimes similar, the development of any one of them will help the others. This work will focus on trapping a single particle in a microfluidic chip.

In recent decades, the methods of manipulating a single particle, droplet, and cell have been quickly developed. Usually, they can be divided into two categories: passive methods and active methods. In the passive method, the particles are often manipulated by the device itself, according to the characteristics of particles or channel geometry. Rambach et al. [7] demonstrated a multi-height device for the single droplet trapping and release. Droplets are passively captured in a microchannel cavity through capillary valve action and released on-demand through a triggered surface acoustic wave pulse. In their device, the droplets can be selected on-demand from an emulsion of continuously flowing droplets and the single droplet can be incubated for a desired duration of time. However, the structure and the fabrication of the chip are very complicated. Moreover, their device cannot capture more than one droplet at the same time. Compared with the active control, the passive control is more casual and needs a larger volume of sample agents. Thus, more active control systems have been recently proposed. The active manipulation method has more advantages in the applications, which can manipulate target particles, droplets, or cells more directly. Dielectrophoresis (DEP) is an important method for single droplet, particle, and cell manipulation [8,9,10,11]. Cole et al. [12] demonstrated a kind of DEP microfluidics that combined the flexibility and programmability of microliter dispensing with the scalability and single-cell sensitivity of flowing droplet microfluidics. Acoustic and optical forces have also been utilized for particle [13,14] and droplet [15,16,17] manipulation. Optical tweezers [18] are a typical tool for single cell manipulation, which use a highly focused laser beam to provide an attractive or repulsive force. Optical tweezers have been particularly successful in studying a variety of biological systems [19]. Chiou et al. [20] used direct optical images to control particles. Recently, another efficient active method called the Anti-Brownian Electrokinetic (ABEL) trap [21,22,23] has been proposed and demonstrated to control micro particles. It uses an optical signal to identify particle location and then provides feedback for the direction of the electrical trapping forces, which is more favorable than laser tweezers. Rahman et al. [24] reported an efficient integrated optofluidic 2D ABEL trap for nanoparticle control. With this device, significantly smaller particles can be trapped and its trapping times can be prolonged when compared to one-dimensional control. However, the ABEL trap is mainly used for molecules and nanoparticles. Furthermore, the fabrication of the chip and the external devices are also complicated, time-consuming, and expensive. In addition, the electric field has also been used in these single-particle active manipulations [25,26,27]. Hart et al. [28] demonstrated a particle trap using a linear eletrodynamic quadrupole. This device has the ability to hold single or many particles along one axis stably under the controlled environment. However, this device cannot pick up a single particle from these linearly arranged particles.

In this work, a microfluidic device was proposed for the capture, manipulation, and release of a single particle. This device utilized liquid-metal filled channels as electrodes to perform the active manipulation of the target particle using electrophoresis (EP) force. The fabrication of this microfluidic device is very simple, cheap, and convenient, which can be helpful for the integration and miniaturization of these micro devices into large-scale microfluidic systems.

## 2. Liquid Metal Based Particle Microtrap

**Figure 1:**
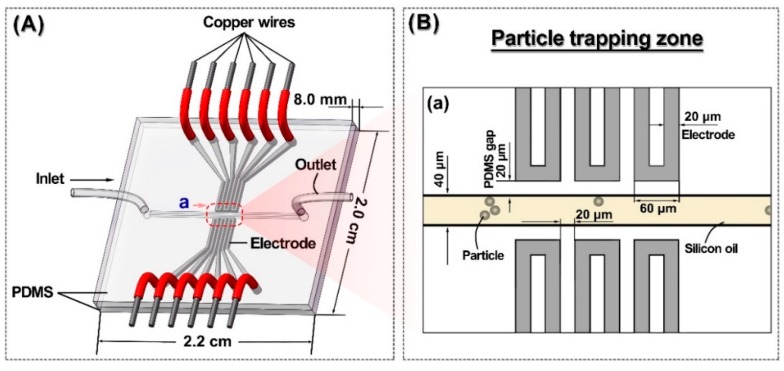
(**A**) Schematic view of a polydimethylsiloxane (PDMS) microfluidic chip; (**B**) Zoom-in schematic of (a). The target particle was trapped under the control of three pairs of liquid metal electrodes.

Figure 1A shows the schematic view of a polydimethylsiloxane (PDMS) microfluidic chip for a particle trap under the electrophoretic force. This chip utilizes liquid-metal filled channels as electrodes to induce an electric field across the 40 μm wide fluid channel. Three pairs of 20 μm wide electrodes were located symmetrically at both sides of the fluid channel in the same horizontal level, and had no contact with the fluid in the channel. The PDMS gap between the fluid channel and the electrode channel was also 20 μm. Figure 1B shows the specific configuration of the trap zone (a) in the chip, and also presents the microscopic image of the particle trap during operation.

## 3. Experimental Details

**Figure 2:**
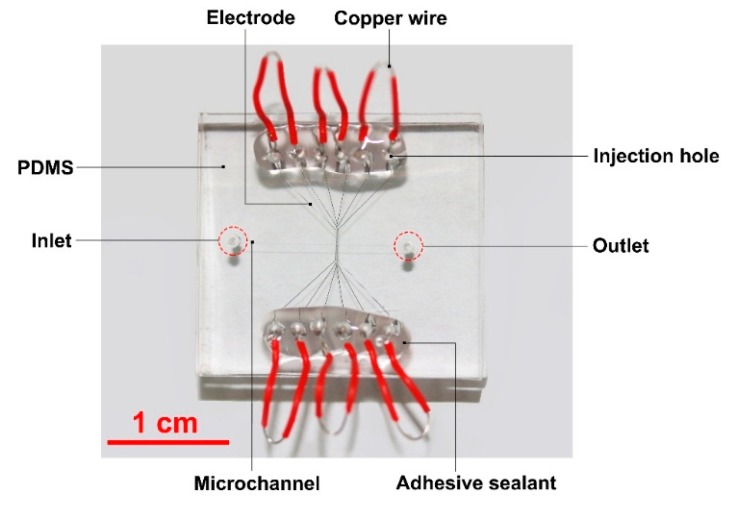
Optical image of a PDMS microfluidic chip for the particle trap though the electric field control induced by the liquid metal electrodes.

Figure 2 shows the optical image of the microfluidic chip used in the experiments. All chips were fabricated using the standard soft-lithography technology. 40 μm high microchannel patterns were fabricated by spin-coating SU8-2050 (MicroChem Corp., Westborough, MA, USA) on a 4 in. silicon wafer. PDMS (mixing base agent and curing agent by 10:1 in weight) was then poured onto the wafer to transfer the channel pattern. The patterned PDMS layer was irreversibly bonded with a PDMS blank layer (2.2 cm × 2.0 cm × 4 mm) by using plasma treatment. Ga_75.5_In_24.5_ (75.5 wt % Ga, 24.5 wt % In, melting point: 15.7 °C; Shanxi Zhaofeng Gallium Co., Ltd., Yangquan, Shanxi, China) was injected into the microchannel to fabricate electrodes using a syringe [29,30]. This method to fabricate the liquid metal electrode in microfluidics for particle manipulations has been used widely in many other works [31,32,33]. Copper wires with a diameter of 200 μm were then inserted into the injection holes at all ends of the liquid metal channels. To ensure a stable connection between the wire and the liquid metal inside the electrode channel, an adhesive sealant (705 RTV Silicone Rubber, Kangda Chemical Co., Ltd., Liyang, Jiangsu, China) was used to seal the wire joints.

**Figure 3:**
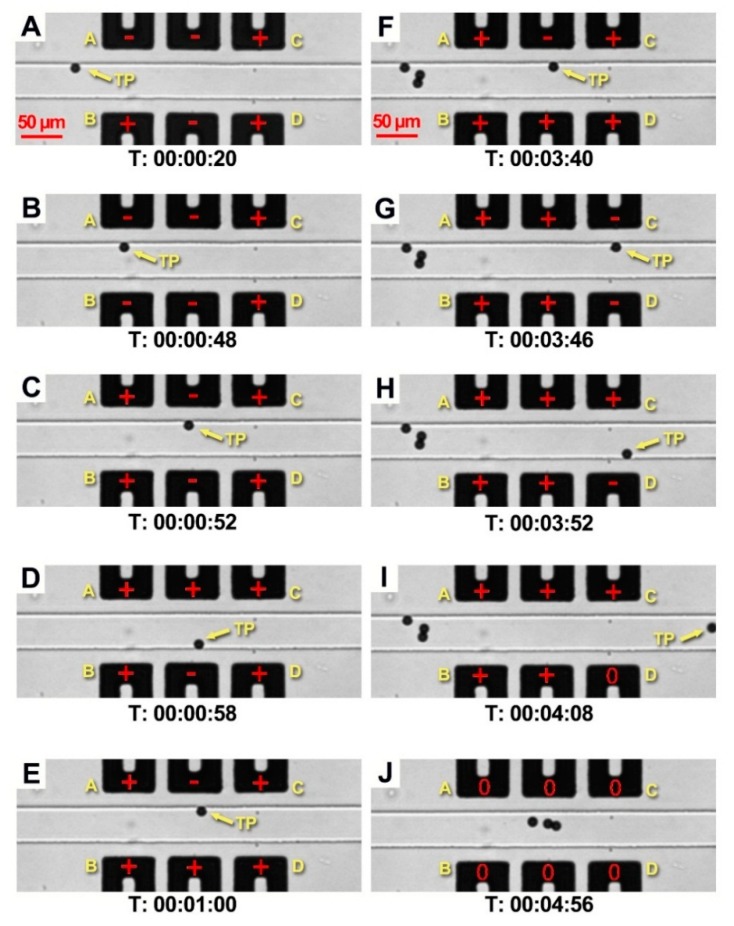
The process of the particle manipulation including: (**A**–**C**) the capture of the target particle (TP); (**D**,**E**) the manipulation of the target particle in the trap; (**F**–**I**) the release of the target particle; (**J**) the release of the other particles. The value of the electric potential applied on the positive electrodes is 700 V, negative electrodes −700 V.

In all experiments, 40 μL fluorescent particles (PE-Cy5, SPHERO^TM^ Fluorescent Particles, 10.5 μm, 1 × 10^7^ particles/mL) were dried at 95 °C on a hotplate, then mixed with 5 mL silicone oil. The sample fluid (silicon oil mixed with particles) was driven by a microfluidic flow control system (MFCSTM-EZ, Fluigent, Villejuif, France), which could control the velocity through a pressure-based flow controller quickly and flexibly when compared to the syringe pump. A direct current (DC) electric field was generated by a high voltage sequencer (HVS448 6000D, LabSmith, Inc., Livermore, CA, USA). The photographs of the channels in Figure 3 were taken by a charge-coupled device (CCD) camera connected to a fluorescence microscope (Axio Observer Z1, Carl Zeiss, Oberkochen, Germany).

## 4. Results

### 4.1. Electric Potential Particle Trap Design

The electrophoretic mobility of the particles suspended in silicon oil can be given by [34]

$$\mu_{e}=\frac{2\varepsilon_{r}\varepsilon_{0}\zeta }{3\eta }$$

where *ε_r_* is the dielectric constant of silicon oil; *ε*_0_ is the permittivity of free space (C²·N^−1^·m^−2^); *η* is dynamic viscosity of silicon oil; and *ζ* is zeta potential. The zeta potential of these particles in silicone oil was positive (6.8 mV, measured by a particle analyzer, Delas^TM^ Nano C, Beckman Coulter, Brea, CA, USA), thus the electrophoretic mobility of the particles in the silicon oil was positive. Therefore, the particles moved from the positive electrode to the negative electrode under the electrophoretic force.

By controlling the layout of the electrode polarity, the target particle can be trapped in the electric field, and at same time, other particles can be blocked outside the trap.

### 4.2. Particle Capture and Release

The steps consisted of the capture and the release of the target particle using the liquid metal electrodes, as shown in Figure 3. During all the experiments, a microfluidic flow control system was used to maintain the pressure of 60 mbar to drive the silicon oil with particles in the fluid channel. The particles suspended in silicon oil moved with the flow at the same flow rate with the silicon oil.

Figure 3 shows the layout of the electrodes. Among the three pairs of electrodes, the first pair can be regarded as the front doors of the trap (Door A and Door B) and the last pair as the rear doors (Door C and Door D). When the target particle moves close to the trap, Door A is opened (switched to −700 V, attracting the target particle) and Door B turns to be closed (700 V, repelling the target particle) (Figure 3A). The target particle slows down and sticks to the top sidewall near Door A. Due to the repulsion of Door B, the target particle cannot get into the trap. Then, as shown in Figure 3B, when both front doors (Door A and Door B) are switched to open, the target particle is attracted into the trap immediately. At the same time, the rear doors (Door C and D) are closed to prevent the target particle from passing through the trap. Then, Doors A and B are closed (Figure 3C) to secure the trapping of the target particle and block the other incoming particles at the same time. With the front doors and rear doors closed, the target particle is trapped at the center of the trap. Figure 3D,E show that the captured target particle can be manipulated in this electric potential trap flexibly by changing the electrode polarity. As shown in Figure 3F, the closed Door A and Door B nicely blocked three other incoming particles from getting inside the trap. Figure 3G–I show that by blocking the other particles outside the trap, the target particle could be selectively released from the trap when the rear doors (Door C and Door D) were opened. When all the electrodes are turned to neutral (Figure 3J), the three blocked particles are allowed to get through the trap with the fluid. Given the limitations of the length of the paper, the whole video (Supplemental Video S1) is included in the Supplementary Materials.

## 5. Discussion

**Figure 4:**
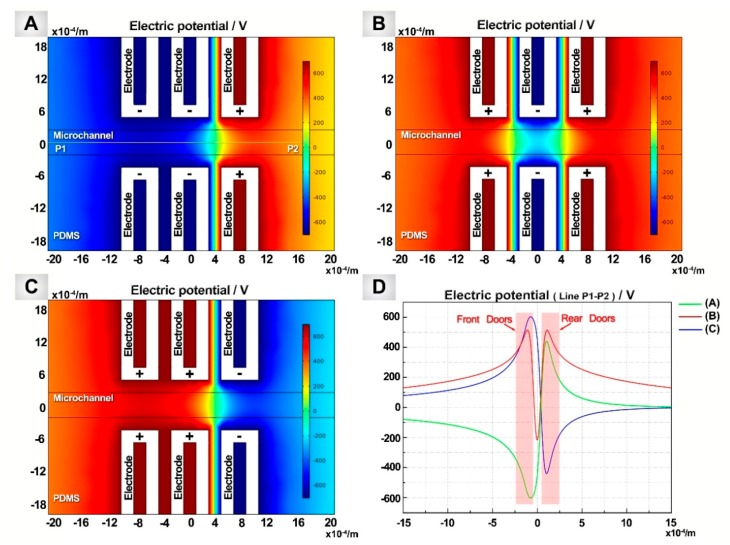
Numerical simulation of the electric potential applied to the electrodes. The value of the electric potential applied on the positive electrodes was 700 V, negative electrodes −700 V. (**D**) The value of the electric potential applied on the central datum line (shown as line P1–P2 in A) of the (**A**–**C**) under the different distributions of electric field.

To explain the mechanism of the particle movement, numerical simulations of the electric field are presented by COMSOL Multiphysics 5.2a (Stockholm, Sweden) (Figure 4). The 3D geometry of the electrodes and the channel patterns is the same as the experimental chips (shown in Figure 1 and Figure 2). The electric potentials of 700 V and −700 V were applied to the electrodes, respectively. The dielectric constants of silicon oil [35] and PDMS [36] were 2.76 and 2.5, and their conductivities were 2.5 × 10^−2^ pS/m and 0.345 pS/m, respectively. Figure 4A shows the electric potential distribution when the front “Doors” are opened. When the target particle flows close to the trap, it will be attracted into the trap because the surface of the particles is positively charged. After the target particle has been captured by the trap, the front “Doors” of the trap are closed (Figure 4B). Figure 4C shows that the back “Doors” of the trap are opened and the target particle is released in this situation. Correspondingly, Figure 4D shows the electric potential distribution along the central line of the fluid channel under Situations A, B, and C.

According to the curves in Figure 4D, when the front “Doors” are closed, the incoming particles are blocked outside the trap unless they can break through the positive electric potential barrier (shown in Figure 4D, curves B and C). For a better understanding of the working principle of the front “Doors”, when a particle is blocked by the front “Doors” and stands still in the flow, the whole process can be explained by utilizing the equilibrium between the pushing electrical force and the flow resistance acting on the particles. According to Stokes’ theorem [37], the flow resistance of a still sphere in the moving fluid can be given as

(1) $$F_{p}=6\pi \mu aU_{m}$$

where $F_{p}$ is the fluid resistance acting on the particle; μ is the dynamic viscosity of the fluid; a is the radius of a sphere; and $U_{m}$ is the fluid velocity. When the external pumping pressure is applied on the fluid in the channel to drive the flow, the relation between the external pressure ∆p and the flow velocity $\;U_{m}$ is given by [38]

(2) $$∆p=f\frac{L}{D_{h}}\frac{\rho U_{m}^{2}}{2}$$

where f is friction coefficient; L is the length of the flow channel; and $D_{h}$ is hydrodynamic diameter, 2wH/(w+H), where w and H are the width and the height of the channel. ρ is the density of the fluid. According to the experimental results, five particles were chosen to calculate their velocities and then their average speed was used to estimate the flow rate of the fluid, and the velocity of the flow was nearly 60 μm/s, the flow rate $5.76\times 10^{-3}$ μL/min, when the external pressure was 60 mbar. For such a flow in the microchannel, the Reynolds number, $Re=\rho U_{m}D_{h}/\mu \approx 6.9\times 10^{-6}$ was far less than 2000. Then, the flow in the microchannel was always a steady laminar flow. So according to [34], f is given by [39]

$$fRe=\frac{64}{G}+\frac{K_{\infty }}{x^{+}},\;x^{+}=\frac{L}{D_{h}Re}\geq 0.1$$

$$G=\frac{2}{3}+\frac{11\alpha \left(2-\alpha \right)}{24},\;K_{\infty }=-0.906\alpha^{2}+1.693\alpha +0.649\;$$

where $x^{+}$ is hydrodynamic entry length; x is the length of the channel; α is channel aspect ratio, w/H. When the fluid channel is 40 μm × 40 μm × 2.0 cm, the density of silicon oil is 969 kg/m^3^, the dynamic viscosity of silicon oil is 0.34 kg/(s·m), the relation between the external pressure ∆p and the flow velocity $\;U_{m}$ can be given by,

$$∆p=1.21\times 10^{8}U_{m}+6.87\times 10^{2}U_{m}^{2}$$

as $U_{m}\ll 1m/s$, the second term of the formula above can be neglected, thus

$$∆p\approx 1.21\times 10^{8}U_{m}\;\propto \;U_{m}$$

According to Equation (1), $U_{m}\propto F_{p}$, thus $∆p\propto F_{p}=F_{EP}$, where $F_{EP}$ is the EP force which is balanced with the flow resistance $F_{p}$. For a particle moving under the EP force, $F_{EP}\propto E$, where E is the electric potential. Thus,

∆p ∝ E

So, to block the incoming particles at the front “Doors”, the minimum applied electric potential should be proportional to the pressure applied on the microchannel. In this paper, the value of the flow pressure was set to 60 mbar and the electric potential was ±700 V.

**Figure 5:**
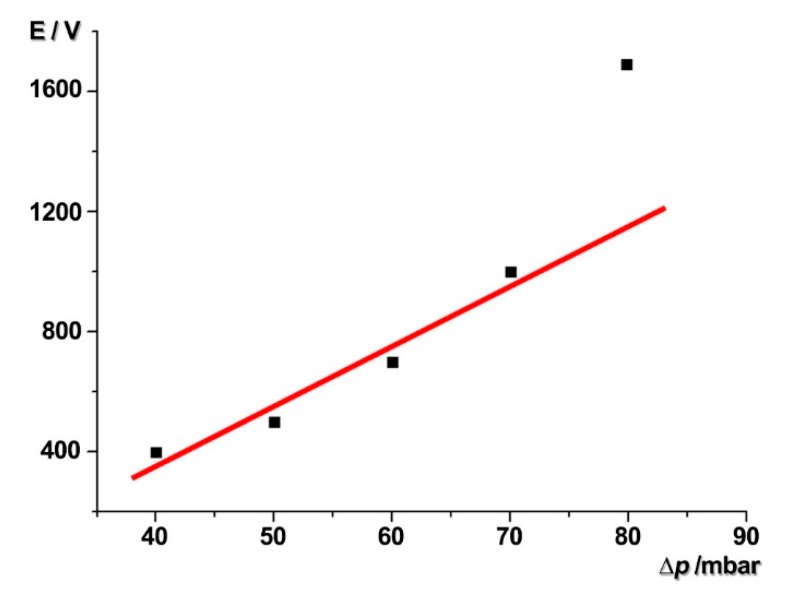
The minimum electric potential applied on the system to stop the particle under different external pressure.

Figure 5 shows the minimum effective electric potential applied on this system under the different external pressures added on the fluid in the microchannel. When the external pressure was less than 70 mbar, the minimum effective electric potential applied on the system was linear with the external pressure, which matched the model and the formula ∆p ∝ E. However, when the external pressure was 80 mbar, the velocity of the fluid was high, and the particle suspended in the fluid also moved quickly. There was not enough buffer distance for the particle trapping due to the inertia of the particle. Then, the particle will pass through the trap before stopping. Thus, a higher value of electric potential is required for the system to guarantee the effect of the particle trapping. The specific explanation (Supplemental Figure S1) is included in the Supplementary Materials.

However, this particle trap had limitations in the aqueous or conductive fluid. According to the concept of the relative permittivity, when an electric field is applied to the medium, an induced charge is generated and the inner electric field is weakened by this induced charge. The ratio of the electric field in the medium to the original applied electric field (in vacuum) is the relative permittivity. As the silicon oil is a non-polar fluid, it had a very small value of relative permittivity (2.76). The weakening effect of the electric field was not great, and the electric potential applied on the particles was much stronger. Thus, the electrophoretic phenomenon was more significant in silicon oil than that in polar fluid. In contrast, the polar fluid, like other aqueous fluids, can weaken the electric field significantly, so the electric potential applied on the particle is weaker. Our additional experiments showed that the particle dispersed in conductive fluid could not be trapped by this structure (data not shown).

This microtrap also had a limitation in manipulating cells. The cells are typically dispersed in a water phase such as the cell culture medium or PBS. Dispersing cells in the silicon oil will decrease the survival of cells, thus this microtrap may not be suitable for cells in most circumstances.

## 6. Conclusions

This work proposed and demonstrated a novel electric potential particle trap using liquid metal electrodes. The particle trap accomplished the task of capturing the target particle and block other ones outside the trap by changing the pattern of the electric potential field. This trapping device has the universality in the active capture and release of a single particle in oil. In our previous work, a similar device was used to control the flow direction of liquid metal droplets [40]. Trapping droplet is also a possible application of this device in the future. However, droplet trapping is much more complicated than particle trapping. Too many factors have to be considered for the droplet trapping, such as shape change of the droplet, droplet size, and changing contact angle. That would go far beyond the scope of this work.

As one of the possible applications of particle trapping in oil, Murali et al. [41] adopted the capacitance counter to monitor the wear debris in lubrication oil to avoid catastrophic system failure of the machine in real-time. This particle trap can be used in a similar case to trap these wear debris and pick out single debris for component detection. Thus, this single particle trap has potential in oil quality testing for picking and detecting mechanical impurities or other insoluble particles in oil.

## Supplementary Materials

The following are available online at http://www.mdpi.com/2072-666X/9/5/221/s1, Supplemental Video S1: Electrophoretic microtrap for single particle. Figure S1: Schematic view of the force analysis of a single particle in the trapper.

## References

[1] Albrecht T., Macpherson J., Magnussen O., Fermin D., Crooks R., Gooding J., Hersbach T., Kanoufi F., Schuhmann W., Bentley C. (2016). Electrochemistry of single nanoparticles: General discussion. Faraday Discuss..

[2] Guo M.T., Rotem A., Heyman J.A., Weitz D.A. (2012). Droplet microfluidics for high-throughput biological assays. Lab Chip.

[3] Teh S.Y., Lin R., Hung L.H., Lee A.P. (2008). Droplet microfluidics. Lab Chip.

[4] Sciambi A., Abate A.R. (2014). Generating electric fields in PDMS microfluidic devices with salt water electrodes. Lab Chip.

[5] Faraghat S.A., Hoettges K.F., Steinbach M.K., van der Veen D.R., Brackenbury W.J., Henslee E.A., Labeed F.H., Hughes M.P. (2017). High-throughput, low-loss, low-cost, and label-free cell separation using electrophysiology-activated cell enrichment. Proc. Natl. Acad. Sci. USA.

[6] Jones P.V., Salmon G.L., Ros A. (2017). Continuous separation of DNA molecules by size using insulator-based dielectrophoresis. Anal. Chem..

[7] Rambach R.W., Linder K., Heymann M., Franke T. (2017). Droplet trapping and fast acoustic release in a multi-height device with steady-state flow. Lab Chip.

[8] Tang S.Y., Zhang W., Baratchi S., Nasabi M., Kalantarzadeh K., Khoshmanesh K. (2013). Modifying Dielectrophoretic Response of Nonviable Yeast Cells by Ionic Surfactant Treatment. Anal. Chem..

[9] Tang S.Y., Yi P., Soffe R., Nahavandi S., Shukla R., Khoshmanesh K. (2015). Using dielectrophoresis to study the dynamic response of single budding yeast cells to Lyticase. Anal. Bioanal. Chem..

[10] Voldman J., Braff R.A., Toner M., Gray M.L., Schmidt M.A. (2001). Holding forces of single-particle dielectrophoretic traps. Biophys. J..

[11] Zhang C., Khoshmanesh K., Mitchell A., Kalantar-Zadeh K. (2010). Dielectrophoresis for manipulation of micro/nano particles in microfluidic systems. Anal. Bioanal. Chem..

[12] Cole R.H., Tang S.Y., Siltanen C.A., Shahi P., Zhang J.Q., Poust S., Gartner Z.J., Abate A.R. (2017). Printed droplet microfluidics for on demand dispensing of picoliter droplets and cells. Proc. Natl. Acad. Sci. USA.

[13] Collins D.J., Alan T., Neild A. (2014). The particle valve: On-demand particle trapping, filtering, and release from a microfabricated polydimethylsiloxane membrane using surface acoustic waves. Appl. Phys. Lett..

[14] Ahmed H., Destgeer G., Park J., Jin H.J., Ahmad R., Park K., Sung H.J. (2017). A pumpless acoustofluidic platform for size-selective concentration and separation of microparticles. Anal. Chem..

[15] Sesen M., Alan T., Neild A. (2014). Microfluidic on-demand droplet merging using surface acoustic waves. Lab Chip.

[16] Jin H.J., Destgeer G., Park J., Ahmed H., Park K., Sung H.J. (2017). On-demand droplet capture and release using microwell-assisted surface acoustic waves. Anal. Chem..

[17] Jin H.J., Lee K.H., Destgeer G., Kang S.L., Cho H., Ha B.H., Sung H.J. (2015). In situ seriate droplet coalescence under an optical force. Microfluid. Nanofluid..

[18] Ashkin A. (1970). Acceleration and trapping of particles by radiation pressure. Phys. Rev. Lett.

[19] Mirsaidov U., Scrimgeour J., Timp W., Beck K., Mir M., Matsudaira P., Timp G. (2008). Live cell lithography: Using optical tweezers to create synthetic tissue. Lab Chip.

[20] Pei Y.C., Ohta A.T., Wu M.C. (2005). Massively parallel manipulation of single cells and microparticles using optical images. Nature.

[21] Dienerowitz M., Heitkamp T., Gottschall T., Limpert J., Borsch M. (2017). Confining Brownian motion of single nanoparticles in an ABELtrap. Proc. SPIE.

[22] Fields A.P., Cohen A.E. (2011). Electrokinetic trapping at the one nanometer limit. Proc. Natl. Acad. Sci. USA.

[23] Thompson M.A., Lew M.D., Moerner W.E. (2012). Extending microscopic resolution with single-molecule imaging and active control. Annu. Rev. Biophys..

[24] Rahman M., Stott M.A., Hawkins A.R., Schmidt H. Design and characterization of integrated 2D ABEL trap. Proceedings of the IEEE Photonics Conference.

[25] Han J.H., Kim H.J., Sudheendra L., Hass E.A., Gee S.J., Hammock B.D., Kennedy I.M. (2013). Electrophoretic build-up of multi nanoparticle array for a highly sensitive immunoassay. Biosens. Bioelectron..

[26] Ramónazcón J., Yasukawa T., Mizutani F. (2011). Sensitive and spatially multiplexed detection system based on dielectrophoretic manipulation of DNA-encoded particles used as immunoreactions platform. Anal. Chem..

[27] Rosales C., Lim K.M. (2005). Numerical comparison between Maxwell stress method and equivalent multipole approach for calculation of the dielectrophoretic force in single-cell traps. Electrophoresis.

[28] Hart M.B., Sivaprakasam V., Czege J., Eversole J.D. Using a Linear Electrodynamic Quadrupole as a Particle Trap. Proceedings of the Optical Trapping Applications.

[29] Gao M., Gui L. (2014). A handy liquid metal based electroosmotic flow pump. Lab Chip.

[30] Gao M., Gui L. (2016). Development of a fast thermal response microfluidic system using liquid metal. J. Micromech. Microeng..

[31] Hallfors N., Khan A., Dickey M.D., Taylor A.M. (2013). Integration of pre-aligned liquid metal electrodes for neural stimulation within a user-friendly microfluidic platform. Lab Chip.

[32] So J.H., Dickey M.D. (2011). Inherently aligned microfluidic electrodes composed of liquid metal. Lab Chip.

[33] Tang S.Y., Zhu J., Sivan V., Gol B., Soffe R., Zhang W., Mitchell A., Khoshmanesh K. (2015). Creation of liquid metal 3D microstructures using dielectrophoresis. Adv. Funct. Mater..

[34] Huckel E. (1924). Die kataphorese der kugel. Physikalische Zeitschrift.

[35] Kuo A.C. (1999). Poly (dimethylsiloxane).

[36] Product Data Sheets. https://www.xiameter.com/en/Products/Pages/ProductDetail.aspxpid=01013084&R=X68EN&C=US#characteristicsAnchor.

[37] Erhard P., Etling D., Muller U., Riedel U., Sreenivasan K.R., Warnatz J. (2010). Prandtl-Essentials of Fluid Mechanics.

[38] Yang X.-H., Tan S., Ding Y., Liu J. (2017). Flow and thermal modeling and optimization of micro/mini-channel heat sink. Appl. Therm. Eng..

[39] Harms T.M., Kazmierczak M.J., Gerner F.M. (1999). Developing convective heat transfer in deep rectangular microchannels. Int. J. Heat Fluid Flow.

[40] Tian L., Gao M., Gui L. (2017). A Microfluidic chip for liquid metal droplet generation and sorting. Micromachines.

[41] Murali S., Jagtiani A.V., Xia X., Carletta J., Jiang Z. (2009). A microfluidic Coulter counting device for metal wear detection in lubrication oil. Rev. Sci. Instrum..

