# Supplementary material for: A Handy Liquid Metal Based Non-Invasive Electrophoretic Particle Microtrap
Source: Micromachines (Basel). 2018 May 7;9(5):221. doi: 10.3390/mi9050221 (PMC6187542; doi:10.3390/mi9050221)
Supplement: Supplementary file 1 [file micromachines-09-00221-s001.zip › micromachines-295059 supplementary material.pdf]

Supplementary Materials: A Handy Liquid Metal Based Non-Invasive Electrophoretic Particle Microtrap

Lu Tian, Lunjia Zhang, Meng Gao, Zhongshan Deng and Lin Gui

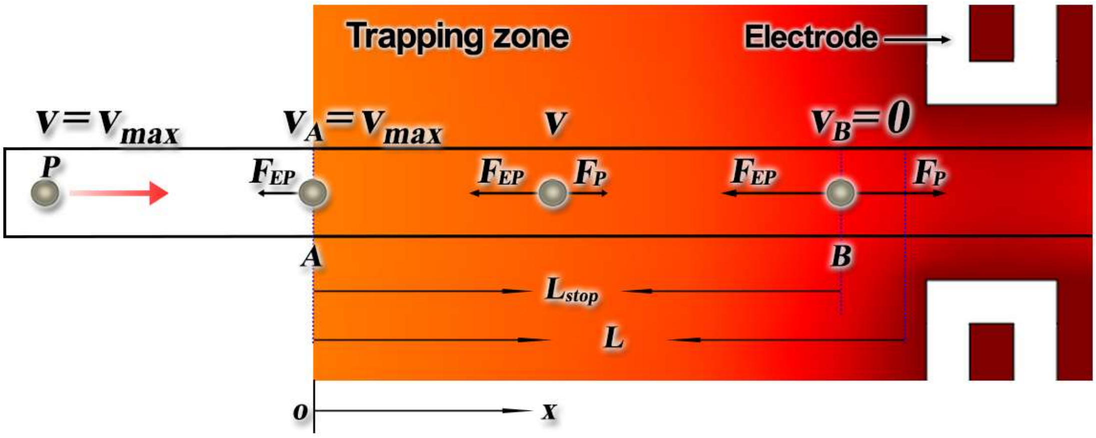

Figure S1. Schematic view of the force analysis of a single particle in the trapper.

Figure S1 shows the schematic view of the force analysis of particle P in the trapper. For particle P, it flows from point A with the velocity of v_{max} (same as the flow) towards the trapper. During the trapping process, two forces are applied on particle P, which are F_p and F_{EP}. F_p is the pushing force from the flow on the particle, and F_{EP} is the electrophoretic (EP) force which is pushing the particle back. As $F_{EP} > F_p$, the particle P will slow down its velocity all the way from A to B and halt at B at the end, where L_{stop} is the distance between point A and point B.

The kinetic energy of the particle P is E_A $(E_A = \frac{1}{2}mv_{max}^2)$ at point A and $E_B(E_B = 0)$ at point B. According to the energy conservation, the trapping process can be expressed as

$$\int_0^{L_{stop}} (F_{EP} - F_p)\mathrm{d}x = E_A - E_B = \frac{1}{2}mv_{max}^2 \tag{S1}$$

$$\int_0^{L_{stop}} F_{EP}dx = \int_0^{L_{stop}} F_P dx + \frac{1}{2}mv_{max}^2 \tag{S2}$$

If particle P can be trapped by this trap, the velocity of the particle must drop to zero before it passes the electrodes, i.e., $L_{stop} \le L$, where L is the permitted maximum distance for particle trapping.

$$\int_0^{L_{stop}} F_{EP}dx \le \int_0^{L} F_{EP}dx \tag{S3}$$

Combining Equation (S2) with Equation (S3), then it can be given as,

$$\int_0^{L} F_{EP}dx \ge \int_0^{L_{stop}} F_P dx + \frac{1}{2}mv_{max}^2 > \frac{1}{2}mv_{max}^2 \tag{S4}$$

$$v_{max} < \sqrt{\frac{2\int_0^L F_{EP}dx}{m}} \tag{S5}$$

As shown in Equation (S5), the v_{max} of the particle must be less than $\sqrt{\frac{2\int_0^L F_{EP}dx}{m}}$. When the external pressure is too high, the balance of Equation (S5) will be broken and the v_{max} will exceed

$\sqrt{\frac{2\int_0^L F_{EP}dx}{m}}$. Then, the particle is impossible to stop in time. Therefore, when the pressure is too high (say, 80 mbar in our case), the only way to stop the particle is to increase $\sqrt{\frac{2\int_0^L F_{EP}dx}{m}}$, or to increase the EP force (we increased the potential to 1700 V in our experiment).
